# Type I interferons in inflammatory bowel diseases: balancing barrier integrity, repair and inflammation in the intestinal epithelium

**DOI:** 10.3389/fmed.2025.1713424

**Published:** 2025-11-25

**Authors:** Mikayla R. King, Roslyn A. Kemp, Safina Gadeock

**Affiliations:** Department of Microbiology and Immunology, University of Otago, Dunedin, New Zealand

**Keywords:** IBD, type 1 interferon, organoid, intestinal epithelium, barrier integrity, clinical response

## Abstract

Inflammatory bowel diseases (IBD), including Crohn’s disease and ulcerative colitis, are persistent, relapsing immune mediated disorders of the gastrointestinal tract associated with significant morbidity and considerable health-care costs. Current therapeutic strategies, such as corticosteroids, immunomodulators, and biologic agents including anti-tumour necrosis factor (anti-TNF) therapies, provide a clinical advantage; however, there is a 30–40% primary non-response rate followed by a 50% secondary non-response rate. This highlights the need for novel therapeutic targets that can specifically address patient heterogeneity. Type I interferons (IFN-Is) are broad acting cytokines that apply state-dependent effects on epithelial intestinal homeostasis. At normal biological levels, IFN-Is reinforce epithelial barrier integrity by regulating tight junction formation, epithelial turnover, and antiviral defence mechanisms. However, in IBD, dysregulated IFN-I signalling facilitates atypical immune cell recruitment, epithelial apoptosis, and prolonged epithelial cell inflammation. The impact of IFN-Is on the dysregulation of the epithelial barrier in IBD remains poorly understood. Patient-derived intestinal organoids represent a physiologically relevant model that aids in the understanding of IFN-I signalling pathways in epithelial cells, allowing for adequate studies on the epithelial–immune crosstalk phenomenon in IBD. This review identifies and highlights IFN-Is as a promising but context-dependent therapeutic target. Interpreting the molecular mechanisms of IFN-Is and their role on the intestinal epithelial barrier function will benefit our understanding of treatment responsiveness as well as aid in resolving current therapeutic challenges in IBD.

## 1 Introduction

Inflammatory Bowel Diseases (IBD) are a group of chronic, relapsing inflammatory diseases of the gastrointestinal (GI) tract, including Crohn’s disease (CD), ulcerative colitis (UC), and indeterminate IBD (1, 2). CD inflammation can occur everywhere throughout the GI tract, from the mouth to the rectum (3). UC inflammation occurs in the colon and rectum, and IC is the term given to patients when the diagnosis is not distinguished between CD and UC (2, 3). CD can present symptoms such as abdominal pain, diarrhoea, weight loss, stenosis, abscesses, fistulas, patchy inflammation, linear ulcers, fissures, ileitis, and granulomas (3). UC is more commonly associated with rectal bleeding, anaemia, superficial ulcers, crypt abscesses, and continuous inflammation of the mucosa, whereas indeterminate colitis symptoms are more variable (3). These symptoms can severely limit the lives of patients with IBD, and with failure to respond to current treatments, IBD can become chronic, leading to relapse (4, 5).

Predicting patient outcomes is challenging due to individualised complex immune responses and no one therapy suits all. Due to patient heterogeneity, genetic variability, microbiome influences, environmental factors, and unreliable biomarker expression, it is difficult to predict response to therapies. Here, we identify Type I IFNs (IFN-I) as a possible new target for biologic therapy and highlight the importance of organoid models to test the effect of IFN-I on the epithelium of people with IBD.

## 2 Current treatments

Therapeutic strategies for IBD include broad anti-inflammatory agents to targeted immunomodulation [reviewed in (6, 7)]. Immunosuppressive therapies such as azathioprine, mercaptopurine, and methotrexate suppress the immune response in patients with moderate to severe IBD (8). Corticosteroids are used to induce remission during acute flares but are unsuitable for long-term use. Immunomodulators and immunosuppressants (azathioprine, 6-mercaptopurine, methotrexate, cyclosporine, tacrolimus) are reserved for steroid-dependent or resistant cases and are often combined with biologics (8). Biologic therapies include anti-tumour necrosis factor (TNF) antibodies (adalimumab, golimumab, infliximab, certolizumab), anti-IL-12/IL-23 and anti-IL-23 antibodies (ustekinumab, risankizumab), which reduce intestinal inflammation (9), and integrin inhibitors (vedolizumab, natalizumab) that interfere with immune cell trafficking and interactions (10). Small molecules such as Janus kinase (JAK) inhibitors (tofacitinib, filgotinib, upadacitinib) prevent phosphorylation of signaling molecules and lead to decreased transcription of inflammatory cytokines (11). Collectively, these therapies protect the integrity of the intestinal epithelial barrier (8).

Anti-TNFs bind to soluble TNF, membrane-bound TNF, and TNFR1/2 receptors to neutralize TNF and downregulate the inflammatory response (12). Anti-TNF therapy costs over $25 billion in the United States per year (13), which puts pressure on patients and clinicians in terms of predicting an appropriate treatment option (4, 14). 30–40% of patients are primary non-responders to anti-TNF and 50% of patients become secondary non-responders (15). There is a need to predict patient responsiveness and question which therapy(ies) would be beneficial for the individual (6, 8, 16).

## 3 The epithelium as a therapeutic target

The key pathogenic feature of IBD is the dysregulation of the epithelial barrier in chronic inflammation (17). The mechanisms that drive epithelial barrier dysfunction in IBD include the disruption of tight junction proteins, increased epithelial cell apoptosis, altered mucus secretion, and increased microbial dysbiosis (17).

The intestinal epithelial cells (IEC) of the GI tract act as a barrier between the immune system in the lamina propria and the microbial environment in the lumen. IEC are generated from stem cells that form a protective barrier comprising goblet cells, enterocytes, Paneth cells, microfold cells (M cells), and colonocytes (18, 19). The role of goblet cells is to secrete mucus to trap microbes in the GI tract (20). Enterocytes produce digestive enzymes and absorb nutrients via microvilli on the apical surface (21). Paneth cells kill microbes via the secretion of antimicrobial peptides (AMP), and enzymes (21). M cells mediate antigen recognition from the lumen to the Peyer’s patches in the lamina propria to initiate an immune response (21, 22). IEC protect the host by aiding in innate signalling factors such as the release of AMP, cytokines, chemokines, and growth factors (20, 22). All these cells of the intestinal epithelial barrier maintain homeostasis via epithelial turnover, apoptosis, and proliferation, supporting a healthy environment within the gut (18, 22, 23).

The GI tract is constantly exposed to antigens from food or microbes, therefore, it is important to have a structurally functional epithelial barrier. The IEC barrier uses leak-and-pore pathways to selectively modulate transport of these components across the barrier which is mainly regulated by tight junction proteins at the intercellular junctions (24). Tight junction proteins aid in the absorption of nutrients, water, the flow of ions, salts, and immune cell signals, while restricting antigen and microbial transportation from the lumen to the lamina propria (21, 22, 25). Structural damage to the IEC can compromise the intestinal epithelial cell barrier, which can lead to increased inflammation and immune cell dysregulation (26).

Altered mucus secretion increases microbial translocation and their derived components across the epithelial barrier leading to immune cell hyperactivation (20, 22, 25, 27). Epithelial cells in people with IBD have altered functional properties and are linked to stem cell dysfunction and inducing progenitor cell abnormalities (23). These epithelial abnormalities are linked to abnormal mucins, hormone, AMPs, defensins, growth factor secretion, and epithelial transport function changes (22, 23). Increased microbial dysbiosis favours the growth and exposure of pathogenic bacteria and the reduction of butyrate producing bacteria disrupting the normal balance of the epithelial lineages (20, 25, 28, 29). These pathogenic bacteria favour immature, proliferating secretory cells as an energy source rather than mature enterocytes (20, 28, 29). Studies using single-cell ribonucleic acid sequencing (sc-RNA-seq) have identified certain epithelial subtypes that contribute to the pathogenesis of IBD inflammation (30–32) - goblet cells, Paneth cells, epithelial transport cells, and secretory proliferative cells alterations lead to increased IBD inflammation (30–32).

Strong evidence from *in vitro* and *in vivo* studies shows that TNF, IFN-*γ*, and IL-1β consistently increase intestinal epithelial permeability, while IL-10 is a protective cytokine, stabilising the barrier, particularly under hyperpermeable conditions (33). Findings for IL-6, IL-17, IL-22, IL-23, and IL-33 are more variable, with both barrier-protective and barrier-disruptive roles reported, reflecting the heterogeneity of cytokine sources and their immune microenvironment. IFN-I add further complexity: while transient IFN-I signalling can support epithelial repair, chronic or excessive activation has been linked to apoptosis, impaired stem cell renewal, and persistent barrier dysfunction (33). A correlation was shown between increased epithelial tight junction permeability and leakiness to increased expression of IL-1β in Caco-2 cells (34). Increased permeability of the intestinal epithelial barrier was associated with reduction of tight junction proteins and an increase in TNF and Type I or II IFNs (35).

It is important to develop therapies that target the main area infected in IBD inflammation - the intestinal epithelial cells, to aid in mucosal healing (8, 16). Intestinal epithelial barrier restoring therapies for IBD are limited and, therefore, there is a need for a better understanding of IBD pathogenesis in epithelial cells for individuals (8, 16).

## 4 IFN-I as a new therapeutic target

Interferons are a group of cytokines comprising IFN-I, type II interferons and type III interferons. Interferons play a critical role in the pathogenesis of IBD inflammation (36). Soluble IFN-I binds to the IFN alpha receptors 1 and 2 (IFNAR1) and (IFNAR2) present on the epithelial basolateral cell surface and this activates downstream signalling pathways to activate the JAK and signal transducer and activator of transcription (STAT) pathway to mediate an immune response (37). The activation of this pathway leads to the phosphorylation and activation of JAK proteins in the epithelial cytosol leading STAT proteins to dimerise and translocate into the nucleus to transcribe interferon-stimulated genes (ISG) to drive epithelial innate immune signalling (37). In homeostasis these ISG act upon immune and epithelial cells via autocrine or paracrine signalling as a response to microbes or cell damage (37). These interferons maintain immune cell signalling and intestinal epithelial cell balance by preventing inappropriate immune cell activation, supporting epithelial cell turnover, and surveilling the luminal microbial-immune interface (36, 38). However, interferons in the context of IBD can be excessively upregulated leading to immune dysregulation and chronic inflammation of the intestinal epithelial cell barrier (39).

The role of type II Interferons such as IFN-*γ* in intestinal epithelial cell signalling in IBD has been well documented (33). An anti-IFN-γ antibody, fontolizumab, was tested in clinical studies in CD patients; however, there was no improved disease outcome (5, 6). Type III IFNs include the IFN-λ subgroup (IFN-λ1-4). IFN-λ and IFNLR are upregulated in IBD patients (40), though whether this has protective or harmful effects remains unclear. IFN-λ signals through a receptor complex composed of IFNLR1 and IL-10R2 (41). IFN-λ exhibit tissue-specific expression, acting mainly on epithelial cells and immune cells (41, 42). This specificity positions IFN-λ as a key player at barrier sites such as the gastrointestinal tract. In mouse models of colitis, type III IFNs limited tissue-damaging neutrophil activity, reducing intestinal inflammation (43).

The role of IFN-I has not been well-studied in intestinal epithelial responses in IBD. Under homeostatic concentrations, IFN-I prime the epithelium to respond to the intestinal microbes and their metabolites by promoting epithelial signalling pathways (36, 38, 44). These pathways include signals such as anti-microbial peptides, defensins, cytokines, growth factors, and chemokines (36, 38). In the context of IBD, however, the pro-inflammatory effects of IFN-I contribute to sustained, dysregulated expression of pro-inflammatory cytokines such as TNF, IL-1β, and IL-6. These cytokines promote epithelial cell apoptosis and enhance the activation of T cells and natural killer cells (38, 45, 46). IFN-I can exert their protective anti-inflammatory effects in early IBD inflammation; however, in later stages of inflammation, IFN-I failed to maintain epithelial restoration (45).

In IBD inflammation, IFN-I are upregulated in response to microbial, injury, and cytokine signals similarly to TNF; it is assumed these signals regulate epithelial cell turnover and mucosal healing (36, 38, 45). Initial IFN-I signals lead to the production of IL-27, IL-1RA, and IL-10, and reduce the expression of proinflammatory cytokines, such as IL1β; however, prolonged IFN-I signalling can enhance chemokine release that increases inflammatory immune cell recruitment, supporting development of inflammation (45). *In vivo* IBD models have illustrated a dual role of IFN-I in IBD. Deletion of IFNAR1/2 resulted in epithelial barrier dysfunction by reducing amphiregulin expression, which is an epithelial proliferation and barrier modulator (47).

While *in vivo* studies suggest a protective role for IFN-I in colitis, the epithelial-specific effects of IFN-I in IBD patients indicate potentially disruptive impacts, including barrier damage, despite some promise shown by IFN-I therapies in clinical trials (36, 48, 49). IFN-β-1a treatment induced clinical improvement and remission in UC patients, associated with a reduction in IL-13 production; elevated IL-17 and IL-6 levels predicted non-response (50), and a study using IFN-β in refractory UC showed high response rates (51). Administration of subcutaneous IFN-β-1a led to remission in 3/10 patients with UC versus none in the placebo group (52). However, a randomized controlled trial involving 91 patients with steroid-refractory UC did not demonstrate significant clinical efficacy (53). Administration of IFN-*α*-2a improved Powell-Tuck disease activity index, IBDQ score, and histology in UC patients, but outcomes did not differ significantly from those achieved with prednisolone (54). A large randomized trial of pegylated IFN-*α* also did not show clinical benefit, although higher doses led to a reduction in C reactive protein levels (55). A 24-week trial of recombinant IFN-α in CD patients did not induce remission or improve inflammatory markers, while a phase II multicentre study of IFN-β-1a showed no benefit over placebo in maintaining steroid-induced remission; both studies were limited by small sample sizes (56, 57).

Studies using human patient samples are essential to clarify how fundamental IFN-I signalling may differ between mouse models and humans, and to identify appropriate experimental systems that more accurately reflect human IFN-I signalling in IBD. The link between IFN-I signalling and TNF responsiveness has not been studied in clinical trials. IFN-I genes are downregulated or restored in patients responsive to anti-TNF therapy to near control levels (48, 49). Other studies have shown that IFN-I genes in immune cells and IEC are upregulated in those patients non-responsive to anti-TNF therapy in IBD inflammatory conditions (48, 49, 58). However, further studies are needed to understand why IFN-I genes are upregulated and what mechanisms are associated that drive the determination of an anti- or pro-inflammatory IFN-I response in IBD inflammation.

## 5 The intestinal epithelium and IFN-I

The intestinal epithelium barrier integrity is maintained and repaired by epithelial cell turnover events that are tightly controlled at homeostasis (17, 59). IFN-I maintain epithelial integrity by promoting epithelial tight junction formation and the restriction of apoptosis via the activation of guanylate binding protein-1 (GBP-1) (60, 61). Deletion of IFNAR1 in mice showed that without the appropriate binding of IFN-I, epithelial cell proliferation and Paneth cell numbers were increased; however, the mice did not develop spontaneous or induced intestinal inflammation (62). IFN-I could also regulate epithelial barrier integrity via the regulation of the cyclin-dependent kinase inhibitor p21 and p53 to control IEC proliferation (63). These results were shown by the deletion of casein kinase 1α, which phosphorylates β-catenin to regulate epithelial WNT signalling and controls IFNAR1 expression (63), in mice.

Epithelial cells play an active role in interferon signalling. Experiments using primary IEC organoids and established cell lines showed that IFN-I strongly induced ISG expression, including *ISG15*, *OAS* and *IFIT* families, and restricted viral replication *ex vivo* (64). While IFN-I activates broad transcriptional programs, they promote antimicrobial defences, early phases of repair, apoptosis-related pathways and epithelial cytotoxicity (64). IFN-I activity in IECs is highly context dependent, balancing protective antiviral defence with potential risks for epithelial injury. Thus, IFN-I can influence epithelial barrier integrity in a context-dependent manner by modulating epithelial cell turnover, highlighting their key role in regulating IEC proliferation and function.

## 6 Organoids as a tool to test IFN-I in IBD

Organoids are an excellent model to understand the physiology and intrinsic regulation of the intestinal epithelium in humans (65). Innate properties of the epithelium can be modelled and diseases can be studied in donor specific patient-derived intestinal organoids (66). The intestinal epithelial cell barrier can be reproduced *in vitro* via organoids derived from patient epithelial crypt basal stem cells acquired after routine biopsies (67). Organoids are three-dimensional structures that develop by the addition of a growth factor enriched culture medium to promote the differentiation from stem cells of Paneth cells, goblet cells, Tuft cells, M cells, and enteroendocrine cells that make up the intestinal epithelial barrier (68–70). Stem cells isolated from intestinal crypts form colonospheres when cultured with media containing epidermal growth factor, noggin, R-spondin, and WNT growth factors along with biomatrix supplementation to develop into mature colonoids (69). Colonoids are crypt-like budding formations reflecting the human physiological epithelial cell types (69). An advantage of using organoids over traditional methods for studying IBD is that the microbiota, mesenchyme, and immune cells of the intestinal epithelial environment can be reproduced to understand how the epithelium responds to complex interactions seen in IBD. This *in vitro* model allows the study of personalised gut microenvironments and patient specific responses (66, 69–71). Previous studies from our lab and others have shown that there are innate epithelial defects in organoids derived from IBD patient colonic biopsies (66, 70, 72, 73). These defects are consistent across the epithelial transcriptome level and metabolome level and are linked to commensal microbial susceptibility (66, 70, 72, 74).

Human intestinal organoids were first generated from pluripotent stem cells (iPSCs) or embryonic stem cells (ESCs) (75, 76). Unlike tissue-restricted stem cells, iPSCs generate all germ layers, producing organoids that develop crypt-like structures and contain multiple IECs, mesenchymal cells, and blood cells (75). However, without further manipulation, organoids from iPSCs retain a fetal-like state, unlike more mature adult stem-cell-derived organoids. Moreover, their induction carries potential epigenetic variations, resulting in differences in structure, function, and genetic features compared with *in vivo* IECs (77). The efficiency of iPSC-derived intestinal organoid generation is currently low, and the technique remains imperfect.

Epithelial models have been central to defining how IFN-I regulate intestinal biology. Studies using cell lines such as Caco-2, HT-29, and T84 and *in vivo* transgenic mouse models targeting epithelial deletions provided tractable systems to dissect signalling pathways (78). These models confirmed activation of the JAK–STAT pathway and were invaluable for identifying ISRE-regulated promoters, mapping apoptosis pathways, and assessing viral restriction, despite lacking the multicellular complexity of organoids (79).

Cell line and *in vivo* studies further established the dual roles of IFN-I in epithelial survival and barrier regulation. Administration of IFN-I prevented epithelial apoptosis in an *Escherichia coli*-induced mouse model (80). Basolateral IFN-*α* polarized murine intestinal epithelial monolayers, protected them from apoptosis, but promoted disruption of epithelial tight junctions (81). These studies show that IFN-I signalling can constrain epithelial proliferation while supporting maturation and differentiation, thereby preserving barrier function and host-microbiota equilibrium.

Intestinal organoids now offer physiologically relevant systems that retain crypt-villus architecture and lineage diversity, enabling more refined dissection of IFN-I biology. Organoid and animal studies demonstrate a context-dependent dual role of IFN-I during epithelial injury: acute signalling enhances antimicrobial defences and promotes repair, whereas prolonged or excessive activation induces growth arrest, impaired proliferation, and chemokine secretion, hallmarks of chronic inflammation (36, 38, 47). This context dependency may explain why IFN-I are protective in some colitis models yet pathogenic in others, with timing, dose, and inflammatory milieu acting as critical determinants of outcome (36). Organoid models have helped define apoptosis as a key mechanism through which IFN-I shapes epithelial function (82, 83). In organoids derived from CD donors, epithelial apoptosis contributed to barrier dysfunction (84). Elevated IFN-I expression in organoids derived from CD donors who were non-responsive to anti-TNF therapy correlated with loss of epithelial integrity, while IFN-I blockade restored barrier function (85).

Organoid platforms incorporating immune cells and single-cell transcriptomics further expand understanding of epithelial IFN-I (86). Immune-derived IFN-I amplified epithelial ISG responses, while epithelial IFN-I modulated immune cell recruitment and activation (87). Sc-RNA-Seq of human intestinal organoids demonstrated heterogeneity in IFN-I responses across epithelial lineages, with Paneth and stem-like cells exhibiting distinct transcriptional programs compared to absorptive enterocytes (62). Comparative studies of murine and human organoids further reveal species-specific differences in ISG breadth and cytokine sensitivity, providing mechanistic explanations for divergent colitis phenotypes across animal models and human disease (33).

## 7 Discussion

Insights from cell lines, mouse models and intestinal organoids underscore the context-dependent nature of IFN-I signalling in the intestinal epithelium. IFN-I pathways span protective antiviral and reparative functions through apoptosis, barrier disruption, and facilitation of pathogen dissemination. Intestinal organoids, with their architectural and lineage fidelity, now represent a powerful platform to disentangle these responses and identify opportunities for therapeutic intervention in IBDs.
